# Experience of sleep disruption in primary Sjögren’s syndrome: A focus group study

**DOI:** 10.1177/0308022617745006

**Published:** 2018-01-11

**Authors:** Katie L Hackett, Vincent Deary, Katherine HO Deane, Julia L Newton, Wan-Fai Ng, Tim Rapley

**Affiliations:** 1 Clinical Academic Occupational Therapist, Musculoskeletal Research Group, Institute of Cellular Medicine, 5994 Newcastle University, UK; 2 Newcastle upon Tyne Hospitals NHS Foundation Trust, UK; 3 Professor of Health Psychology, Department of Psychology, Northumbria University, UK; 4 Senior Lecturer, School of Health Sciences, University of East Anglia, UK; 5 Clinical Professor of Ageing & Medicine, Institute of Cellular Medicine, 5994 Newcastle University, UK; 6 Professor of Rheumatology, Musculoskeletal Research Group, Institute of Cellular Medicine, 5994 Newcastle University, UK; 7 NIHR Newcastle Biomedical Research Centre, 5994 Newcastle University, UK; 8 Professor of Applied Health Care Research, Department of Social Work, Education & Community Wellbeing, Northumbria University, UK

**Keywords:** Sleep, long-term conditions, qualitative research

## Abstract

**Introduction:**

Primary Sjögren’s syndrome is the third most common systemic autoimmune rheumatic disease, following rheumatoid arthritis and systemic lupus erythematosus, and results in dryness, fatigue, discomfort and sleep disturbances. Sleep is relatively unexplored in primary Sjögren’s syndrome. We investigated the experiences of sleep disturbances from the viewpoint of primary Sjögren’s syndrome patients and their partners and explored the acceptability of cognitive behavioural therapy for insomnia.

**Method:**

We used focus groups to collect qualitative data from 10 patients with primary Sjögren’s syndrome and three partners of patients. The data were recorded, transcribed verbatim and analysed using thematic analysis.

**Results:**

Five themes emerged from the data: (a) Experience of sleep disturbances; (b) variation and inconsistency in sleep disturbances; (c) the domino effect of primary Sjögren’s syndrome symptoms; (d) strategies to manage sleep; (e) acceptability of evidence-based techniques. Sleep disturbances were problematic for all patients, but specific disturbances varied between participants. These included prolonged sleep onset time and frequent night awakenings and were aggravated by pain and discomfort. Patients deployed a range of strategies to try and self-manage. Cognitive behavioural therapy for insomnia was seen as an acceptable intervention, as long as a rationale for its use is given and it is tailored for primary Sjögren’s syndrome.

**Conclusion:**

Primary Sjögren’s syndrome patients described a range of sleep disturbances. Applying tailored, evidence-based sleep therapy interventions may improve sleep, severity of other primary Sjögren’s syndrome symptoms and functional ability.

## Introduction

Primary Sjögren’s syndrome (PSS) is the third most common systemic autoimmune rheumatic disease, following rheumatoid arthritis and systemic lupus erythematosus (Yu et al., 2013). It is a connective tissue disease usually diagnosed and treated by rheumatologists. PSS affects predominantly women and is most commonly diagnosed in the fifth decade of life (Qin et al., 2015). The disease process results in dryness, particularly of the eyes and mouth. Dryness is managed with topical pharmacological treatments and specialist medical and dental care (Price et al., 2017). Other known features of PSS include chronic fatigue (Ng and Bowman, 2010) and chronic widespread pain (Segal et al., 2013).

Our previous work demonstrated that people with PSS experience difficulty performing functional activities (Hackett et al., 2012). Consequently, we undertook a study to identify priority intervention targets to improve occupational performance in PSS patients (Hackett et al., 2014). This work demonstrated that poor sleep is a priority intervention target (Hackett, in press; Hackett et al., in press). We conducted a systematic review exploring the prevalence of sleep disturbances in PSS and found that sleep disturbances, particularly night awakenings, are problematic for many PSS patients (Hackett et al., 2017), adding to the overall disease burden.

Sleep disruption in PSS is likely to interfere with aspects of occupational performance and impact upon daytime fatigue. Fatigue and daytime sleepiness are reported as separate symptoms, but there is likely to be a high degree of overlap between these two features. There are few published studies exploring fatigue and sleep disturbances and their impact directly with PSS patients. We previously found a relationship between reduced daily function and both daytime sleepiness and fatigue (Hackett et al., 2012). Another group found that discomfort during the evening would often result in a disturbed night’s sleep (Goodchild et al., 2010). Another recent qualitative study found that discomfort associated with oral dryness could result in disturbed sleep (Lackner et al., 2017) in PSS patients.

Sleep disruption in older adults impacts on quality of life, health and occupational engagement (Leland et al., 2016). Interestingly, the American Occupational Therapy Association (AOTA) recognises sleep as a core occupation, and further, call upon occupational therapists to support their patients with sleep hygiene to improve daily function (American Occupational Therapy Association, 2014). However, cognitive behavioural therapy for insomnia (CBT-I) (which includes a sleep hygiene education component) is more effective than sleep hygiene advice alone in people with chronic insomnia at improving sleep onset latency, wake after sleep onset, total sleep time and sleep efficiency (Trauer et al., 2015). Furthermore, it is an effective treatment for chronic insomnia in a range of long-term conditions (Morgan et al., 2012). Therefore, occupational therapists should consider using the best available evidence, supported by appropriate training within their practice, to improve both the occupation of sleep itself and participation in daytime occupations.

A CBT-I intervention typically includes several components. These include education (sleep hygiene), behavioural components (such as stimulus control and sleep restriction therapy) and cognitive therapy around unhelpful thoughts and beliefs about sleep (Perlis et al., 2005). Sleep hygiene education includes instructions about the sleep environment and sleep habits such as avoiding daytime naps, advice, and limiting alcohol, caffeine and nicotine intake before bed (Trauer et al., 2015). Stimulus control therapy aims to strengthen the association of the bed/bedroom with sleep and weaken associations between the bed/bedroom and activities (other than sex) which interfere with sleep (Bootzin et al., 1991). Sleep restriction therapy (Spielman et al., 1987) involves restricting time in bed and agreeing to retiring and wake-up times following the completion of a sleep diary. Sleep restriction therapy is thought to increase the drive to sleep, stabilise circadian control of sleep and wakefulness, and reduce (hyper)arousal prior to sleep, which leads to reduced sleep onset latency and a more consolidated, uninterrupted sleep (Kyle et al., 2014). Best practice in CBT-I recommends the collection of a 2-week sleep log to identify patterns of sleep–wake times and variability prior to initiation of sleep restriction therapy (Schutte-Rodin et al., 2008).

Despite the evidence of CBT-I in chronic insomnia and a range of long-term conditions, to our knowledge, there have been no published randomised controlled trials investigating its effectiveness in people with PSS. Therefore, little is known about the acceptability and efficacy of CBT-I in PSS patients.

## Aim

The first aim of this study was to explore sleep with people with PSS and their partners and the impact of sleep disturbance on their daily lives. Second, we sought to explore potential feasibility and acceptability of interventions addressing sleep disturbances.

## Method

We used focus groups to collect qualitative data. The interaction involved in focus groups means that participants are encouraged to talk with each other, thus enabling them to comment on each other’s experiences and viewpoints, whilst being able to ask each other questions and provide anecdotes (Kitzinger, 1995). Focus groups are an appropriate means to explore a subject, as participants are able to discuss the topic with each other and focus on issues they perceive as being important, using their own vocabulary (Kitzinger, 1995). Furthermore, focus groups can be used in the development phase of complex intervention development, particularly if a systematic review of the existing literature has not answered questions about the effectiveness or acceptability of existing interventions (Craig et al., 2006).

### Participants

We invited 62 potential adult participants to take part in this study, 44 of whom were PSS patients who were members of the United Kingdom Primary Sjögren’s Syndrome Registry (UKPSSR) (Ng et al., 2011), thus fulfilling the American European Consensus Criteria (Vitali et al., 2002). This ensured that all patient participants had a clinical diagnosis of PSS. The remaining 18 people we invited to take part were adults living in the same household as someone with PSS. All potential participants had taken part in a previous mixed methods study (Hackett et al., 2014) and the patients attended a specialist PSS clinic led by one of the co-authors (W-FN). Timings of the groups were planned around the availability of facilitators and participants. Participants provided informed consent and signed consent forms prior to taking part in the focus groups. Ethics approval was granted by granted by the Office for Research Ethics Committees Northern Ireland (13/NI/0190, IRAS Ref: 125562) and the study was registered on the National Institute of Health Research Comprehensive Clinical Research Network’s portfolio of non-commercial clinical research studies (Study ID: 15939).

### Focus groups

The focus groups were all led by the same moderator (KLH), a clinical academic occupational therapist. They were co-facilitated by a clinical academic health psychologist (VD). Both facilitators work clinically in a National Health Service (NHS) fatigue clinic. KLH had met several participants through the course of her occupational therapy clinical work and during a previous research study. VD had not met any of the participants prior to this study. Three 2-hour focus groups were organised to discuss a range of symptoms, their impact and a range of potential intervention components, and participants were invited to all three groups. The focus group sessions included a break and were divided into six separate topics (sleep, fatigue, pain, depression, anxiety and potential modes of delivering future therapy interventions). Two topics were discussed at each focus group, and sleep and fatigue were the topics for the first meeting. However, participants were free to talk about their sleep disturbances and potential interventions for sleep disruption during any of these sessions. The focus groups took part in a meeting room within an NHS outpatient clinic. Some participants had previously attended this clinic for treatment. Whilst we have focused on sleep in this paper, we intend to publish other aspects elsewhere.

At the start of a session, the topic was introduced. The specific session on the topic of sleep was guided by the topic guide (Table 1), which included some lay descriptions of CBT-I intervention components which were expanded upon in a verbal description by the facilitators. We used the questions in the topic guide as a means to spark discussion amongst participants. At the end of each focus group, KLH summarised the conversations as an informal way of checking the interpretation of the conversations with the group members. The meetings were audio recorded and the recordings were transcribed verbatim.

**Table 1.** Sleep topic guide for focus group.

Topic guide
Do you (or your relative) experience any difficulties with sleep?
What are they?
How do you (or your relative) currently manage your sleep difficulties?
Here are some possible non-drug intervention solutions.
What do you think about each? (A brief description was given for each)
• Cognitive behavioural therapy for insomnia (CBT-I)
• Establishing a bedtime wind-down routine
• Removing all activities such as computers/crafts/books from the bed
• Restricting time spent in bed
• Keeping a record of sleep in a sleep diary
• Talking through any unhelpful thoughts about sleep with a healthcare professional
• Getting up after 15 minutes if you (or your relative) haven’t managed to fall asleep

### Data analysis

Thematic analysis was used to analyse the data (Braun and Clarke, 2006). This involved six stages: (a) familiarisation with the data by listening to the audio recordings, reading the transcripts and making notes; (b) generation of initial codes from the data, which were applied to sections of text; (c) examination of the codes and grouping similar meaning codes together, which resulted in the generation of higher order themes; (d) review of these themes in relation to the coded extracts of text to ensure that they worked within the context of the whole dataset. These themes and the codes within them were initially reviewed by a second researcher (TR), followed by the remaining authors; (e) further analysis and refinement of the themes following feedback from other authors; and (f) writing the analysis.

## Results

Postal responses were received from 27 out of the 62 patients and household members (44% response rate). Nineteen respondents indicated that they would like to take part in a focus group. Thirteen were able to attend one or more focus groups, including 10 patients with PSS (eight female and two male) and three partners of patients (two female and one male). Table 2 provides demographic information about the participants. Participants are referred to by either PP (patient participant) or AHM (adult household member). The groups are coded according to the session number; for example, FG1A is the first half of focus group 1 and FG3B is the second half of the third focus group. Line numbers from the transcripts are referenced following the focus group code.

**Table 2.** Demographics of focus group participants.

Participants		Gender	Age (years)	Employment status	Attend FG1	Attend FG2	Attend FG3	Years since diagnosis	Pain (0–100)^a^	Fatigue (0–100)^a^	Dryness (0–100)^a^
PP1	Patient	M	62	Unemployed	Yes	Yes	No	4	49	53	69
PP2	Patient	F	73	Retired	Yes	No	No	26	34	72	58
PP3	Patient	F	46	Unemployed	Yes	Yes	Yes	10	16	96	49
PP4	Patient	F	74	Retired	Yes	No	No	11	23	82	69
PP5	Patient	M	59	Emp P/T	Yes	Yes	Yes	4	12	39	11
PP6	Patient	F	54	Unemployed	Yes	Yes	Yes	2	82	90	37
PP7	Patient	F	65	Emp P/T	Yes	Yes	Yes	7	16	28	33
PP8	Patient	F	77	Retired	No	Yes	Yes	12	23	93	66
PP9	Patient	F	75	Retired	No	Yes	Yes	31	23	29	45
PP10	Patient	F	68	Retired	No	No	Yes	4	30	74	59
AHM1	Spouse	F	66	Retired	Yes	Yes	No				
AHM2	Spouse	M	60	Retired	No	Yes	Yes				
AHM3	Spouse	M	77	Retired	No	Yes	Yes				
Mean (SD)			66 (10)		Total 8	Total 10	Total 9	12 (10)	28 (21)	66 (26)	50 (19)

Emp P/T: employed part-time; FG: focus group

a Visual analogue scale

Five themes emerged from the codes following data analysis, and we discuss them in the following section. The themes were: (a) the experience of sleep disturbances; (b) variation and inconsistency in sleep disturbances; (c) the domino effect of PSS symptoms; (d) strategies used to manage sleep; and (e) the acceptability of evidence-based techniques.

### The experience of sleep disturbances

Sleep was regarded as a precious resource. It was something that was not taken for granted. A full night’s sleep was seen as ‘something to look forward to’ (FG1A PP3 80), despite being a rare experience. Patient participants all reported experiencing sleep disturbances which post-dated the onset of their PSS. For example, someone described previously being able to ‘sleep on a washing line’ (PP7 FG1A 892), indicating that sleep had been relatively effortless. However, following disease onset, she had noted a reduction in sleep quality and an increase in the effort which she needed to put into her sleeping environment to help sleep come more easily.

Other participants also identified with this story. For example, one lady similarly noticed a gradual deterioration in her sleep following her diagnosis.

PP6 It’s a strange thing that all of a sudden it [poor sleep] just creeps up very, very gradually and you think, ‘Oh, this is odd’. (FG1A 173–174)

Only some participants had previously considered their disease as being a possible reason for their sleep problems, but through the focus group discussions, others in the group also considered that their sleep had become worse over time, due to the effects of their PSS. These patients relied on their lay reasoning to make sense of the impact of the disease on their sleep.

PP3 The thing is it affects you so much during the day, realistically when you think about it, why would it switch itself off at night when you went to bed to sleep? (FG1A 722–723)

Given that PSS is ‘with you 24/7’ (PP3 FG1A 754), they felt that it would naturally also impact on sleep. However, not everyone had made this association. Notably, their sleep disturbance also had broader social and relationship consequences. For example, one person said, ‘I’ve thought about saying to my other half, “we’ll have separate rooms”. We already have separate beds’ (PP6 FG1A 116–118). Therefore, the disease can influence sleep quality for those with PSS as well as their bed partners.

### Variation and inconsistency in sleep disturbances

Participants discussed a range of sleep disturbances. Unrefreshing sleep was commonly experienced and one patient, even if she had slept through the night, would ‘wake up and feel as if [she’d] never been to bed’ (PP4 FG1A 247). Another felt as though he had done a ‘night shift as opposed to having a sleep’ (PP5 FG1A 449) due to feeling so exhausted when he woke up. This non-restorative sleep was seen as an ‘emotional disappointment’ (PP7 FG1A 451) due to having an expectation of feeling refreshed in the morning, only to find that this need remained unmet.

Difficulty with sleep onset was also a distinct problem raised. A participant explained that although occasionally he was able to get to sleep relatively quickly, he regularly struggled to get off to sleep and would lie awake during the night thinking ‘When am I going to go to sleep?’ (PP5 FG1A 410). Alongside this problem, people reported regular night awakenings. One lady often found herself awake for ‘two to three hours’ (PP4 FG1A 257) in the middle of the night before falling asleep again and consequently would wake up feeling unrefreshed. Similarly, another participant would eventually doze off after waking during the night. However, she described the sleep she experienced following a long spell of lying in bed awake as ‘not exactly sleep, sleep’ (PP7 FG1A 238) and that this (dozing) sleep was dissatisfying and inferior to regular sleep.

Although participants may experience a particular sleep problem at one time, these can change. For example, a particular problem, such as difficulty with sleep onset, may not consistently be present, but it can be replaced by another sleep disturbance instead. One participant explained that:

PP2 I still have erratic sleep pattern.… I can go to bed at 11 o’clock and not get to sleep at all.… I can go to bed at 11 o’clock and sleep until about 2, and that’s it.… And occasionally, very, very, very occasionally I sleep right through. (FG1A 67–76)

Interestingly, one person shared how his problems were different to those described by other participants with PSS. He did not experience sleep onset difficulties or regular night awakenings. However, he regularly experienced what he considered a short sleep duration. ‘If I get four hours sleep/five hours sleep a night I, I’ll be lucky’ (PP1 FG1A 33). As well as experiencing a short sleep duration, he also gave the impression of an altered circadian rhythm (altered body clock) as he regularly went to sleep at 7.30 pm and woke up at 1.00am.

### The domino effect of PSS symptoms

Patients explained the impact of and interrelationship between other PSS symptoms and their sleep. Oral and ocular dryness in particular caused a huge amount of discomfort for some and they felt it was responsible for waking them in the night:

PP3 I wake up like three of four times, like somebody’s put a hairdryer in your mouth.… And sometimes it catches your throat and you can wake yourself up trying to swallow.… It’s a horrible feeling. And as much as you try to get a glass of water, a second later it’s still like you’ve been in the desert.… And then the dryness, I put Lacri-lube in my eyes, and I can put it in three or four times during the night and I’ll still wake up like they’ve got sand in them. (FG1A 294–315)

Discomfort in the legs was another factor that could interfere with sleep. The various types of leg discomfort described related specifically to poor peripheral circulation and reduced sensation. Pain also disrupted sleep for some people. However, it was unclear whether the pain was due to their PSS or to co-morbid musculoskeletal problems. For example, one participant noted that: ‘Two nights ago I never slept all night ‘cause of the [neck] pain and it had, it went down me arms and me hands’. (PP10 FG3A 193–194).

Sleep disturbances also had consequences for other PSS symptoms, making them seem worse. Fatigue was a natural consequence of poor sleep, which meant the ability to participate and perform daily activities was restricted.

PP4 Yes, you could see, erm, sort of, link too isn’t there between disturbed sleep or inadequate sleep and fatigue the next day so you can’t do the things you want to do during the day. (FG1A 764–766)

However, frustratingly, fatigue did not always lead to sleep at night, and one participant highlighted a reason for this paradox was because ‘the fatigue is a different thing to tiredness’ (PP3 FG1A 392). In this way, sleep was considered a linchpin, as it was seen as a strong influencing factor on other symptoms such as fatigue and depression: ‘If that one’s wrong [sleep] a lot of the other ones’ll fall. I suppose it’s like… the domino theory’. (PP4 FG1B 821–822). If they did not sleep well, they would feel fatigued, consequently feel low in mood and experience more worry and pain. Combined, these symptoms greatly impacted the ability to perform daily activities.

Co-morbidities further contributed to sleep difficulties. For example, one lady who was menopausal and had hypothyroid problems explained:

PP6 The thyroid’s upset if the Sjögren’s is upset and they’ll upset each other. If I’ve got the flushes and the weird mood-swings and whatever’s going on up here in the head, then I start having nightmares and disturbed sleep. (FG1A 124–127)

If one condition was symptomatic, it would affect the others. This resulted in further sleep difficulties and created a vicious cycle.

Unsurprisingly, another impact of sleep disturbances was daytime sleepiness. A patient’s wife described how she regularly observed her husband falling asleep inadvertently during the day:

AHM1 And he’ll be sitting on a chair and then I’ll be talking to him and I’ll [say], ‘Are you listening?’ and he’s asleep, you know, during the day he’s nodded off 10 minutes, and I’m talking away to meself. (FG1A 558–560)

Others also had unplanned naps during the day, such as while sitting down to watch television in the afternoon. Inadvertently falling asleep during the day whilst in the company of other people could be embarrassing at times.

PP5 I was actually at a friend’s yesterday afternoon, we’re chatting, and I actually fell asleep.

PP8 Mid-conversation?

PP5 Yeah, literally, almost. (FG3A 975–982)

One lady fell asleep during a focus group discussion despite being actively involved in the discussion before and after she dozed off. Daytime sleepiness was therefore both observed and described as being a real problem, which clearly impacted on patients’ daily function and participation.

### Strategies used to manage sleep

One participant had resigned himself to having poor sleep for the rest of his life: ‘It’s just something you live with; you can’t do anything else’ (PP1 FG1A 50). However, other people had implemented their own approaches to try to improve their sleep. For example, a daytime ‘powernap’ (PP7 FG1B 749) was one such strategy that patients used to help manage daytime sleepiness and fatigue. Daytime napping offered refreshment, which was not always experienced following nighttime sleep. However, not everyone was able to sleep during the day despite a poor night’s sleep and one participant in particular described experiencing greater fatigue because of her inability to nap.

Participants had also sought help from a range of external sources. For example, some had been prescribed sleeping pills by their GPs. However, none had opted to use them. One participant thought that hypnotic medication might be addictive and she did not want to become reliant on such a drug. Another said, ‘I want control of me, not drugs’ (PP2 FG1A 1114). A few people had tried alternative medicines, including Chinese herbs, Shiatsu and acupuncture. One participant who regularly had acupuncture for pain explained the positive impact on her sleep the night following an acupuncture session: ‘I sleep like a baby. It’s a wonderful, wonderful deep restorative sleep, and I can go all the way through’ (PP6 FG1A 139–141).

Relaxation or meditation techniques to help sleep were an avenue which had been explored by some. One patient told the group how she regularly enjoyed using meditation and relaxation tracks to relax at bedtime. However, despite noticing some benefits, she could not always fall asleep whilst listening to them. Another found that relaxation helped her to ‘calm down and take [her] mind off… worrying things or churning thoughts’ (PP4 FG1A 1649–1653). Therefore, although these techniques did not necessarily induce sleep, they were a positive diversion from anxieties and promoted a sense of wellbeing. Others reported using a radio or television as a distraction to help them sleep.

Patients addressed nighttime discomfort in a variety of ways. One had alleviated the discomfort she experienced in her legs by propping up the end of her mattress in the hope that she would sleep better. Feeling cold at night in bed meant that some people took a great deal of care ensuring that their sleep environment was warm and stayed warm, using electric blankets, hot water bottles and extra blankets. However, these strategies could again affect a bed partner. One patient’s wife described how her husband’s use of hot water bottles in the bed was ‘a bone of contention’ (AHM1 FG1A 896) as she liked to remain cool in bed.

### The acceptability of evidence-based techniques (CBT-I)

The CBT-I components were explained briefly to participants (Table 1). One person had previously received individual CBT-I over several weeks. Sleep diaries are used in CBT-I to measure outcomes and monitor sleep efficiency (the percentage of time spent in bed being asleep). Patient participants who had previously attended the fatigue clinic had been asked to complete sleep diaries. Although the purpose of asking patients to complete these was to measure sleep outcomes and plan treatment, it had surprising benefits:

PP6 That sleep diary, er, that I’ve done… that was incredible. That really made me think, ‘cause… we’re trying to find answers about patterns and all this…. And I think all of a sudden when you start to… log your sleep and… you focus on it… it sort of raised lots of things that I thought, ‘Ah, maybe there are some things I could do…’. I found that sleep diary very, very useful. (FG1A 654–665)

This patient found that by self-monitoring her sleep for a couple of weeks, she became aware that her sleep problems were part of her disease.

The participant who had undertaken CBT-I had been advised to remove clutter and activities from her bedroom as part of this intervention and continued to apply this advice following the completion of her therapy. Another participant, who was hearing this advice for the first time during the focus group, started to make immediate plans to make changes to her bedroom environment when she returned home. Completing the sleep diary and having access to this information was enough for her to consider making changes to her bedroom environment and sleep behaviours.

Although other participants had not formally experienced CBT-I, a few participants recognised some components, as they had previously tried to implement them themselves. Anchoring the wake-up time in the morning (ensuring it is the same time every day) was one such strategy, which had been implemented by some people. Others indicated that they would be willing to give this a go. However, one participant had a different reaction. He said he would ‘find it difficult fixing a time because it depends on the sort of night [he’d] had’ (PP5 FG1A 1447–1448). He explained:

PP5 If you do eventually get to sleep what’s the point of deliberately waking yourself up? You’re having some sleep, thank God. Let me, let me keep it. (FG1A 1676–1678)

However, after being given the rationale behind some of the intervention components, he said that he could see the reasoning behind it, but if he were to try these techniques, he would have ‘to see some, erm, success’ (PP5 FG1A 1844–1845) after implementing any such strategies to motivate him to continue with the treatment.

Concerning sleep onset difficulties, an intervention component recommended to patients within CBT-I is that is that they get out of bed after 15–20 min if they have not fallen asleep. Participants generally considered this advice as being acceptable and two patients said that they already did this, one of whom had previously undertaken CBT-I.

Sleep restriction received a mixed response from participants. Along with anchoring wake-up times, this concept initially seemed counterintuitive. Therefore, participants again felt that it was essential for clinicians to provide a good rationale for any suggested intervention techniques. Following discussion with the facilitators, the group understood that restricting time in bed was to support the regulation of the sleep and for the body to begin to associate being in bed with being asleep, not being awake. It was acknowledged that ‘the patient too has to be willing to experiment’ (PP4 FG1A 1830) in order to see some improvements in their sleep. However, participants would like to see improvements in their sleep relatively quickly following the application of the techniques.

## Discussion

Participants described various types of sleep problems, including unrefreshing sleep, night awakenings, sleep onset difficulties, altered circadian rhythm, and nighttime pain and discomfort. Sleep disturbances are seen in a range of chronic diseases (Kamath et al., 2015) and there are broad similarities between the sleep difficulties experienced in PSS and in other conditions, such as primary insomnia (Reynolds et al., 1991) and chronic pain (Tang et al., 2015). Not all participants had made an association between their sleep disturbances and their PSS. This is possibly due to the prevalence of sleep disturbances, until recently, being relatively under-recognised in PSS (Hackett et al., 2017). Consequently, health care professionals may not raise the topic of sleep during consultations with patients.

Participants perceived that some of their other symptoms interrupted their sleep, including pain, discomfort and dryness. Specific sleep problems in PSS include difficulty with sleep onset, night awakenings, altered circadian rhythm, dryness and short sleep duration (Hackett et al., 2017). In this focus group study, patients’ leg discomfort and altered sensation were specific symptoms interfering with sleep. It is possible that these may be due to peripheral neuropathy or restless leg syndrome, which both occur within PSS populations (Carvajal Alegria et al., 2016; Hening and Caivano, 2008) and may require medical care. Furthermore, if a primary sleep disorder is suspected (such as obstructive sleep apnoea), patients should be referred to the appropriate specialist for further investigations (Hackett et al., 2017).

Night awakenings were a particular problem and they have been reported in this patient group previously (Hackett et al., 2017). Both night awakenings and sleep onset difficulties are symptoms of insomnia, and as such should be amenable to CBT-I (Trauer et al., 2015). However, due to the unique feature of oral and ocular dryness also interfering with sleep in PSS, a dryness management component may form a useful adjunct to a CBT-I intervention.

Participants in this study described an overlap between their sleep and fatigue symptoms. This is neither surprising nor unique to PSS, as this overlap is recognised in both primary insomnia and in other fatiguing conditions (Buysse, 2013; Gotts et al., 2016). They did, however, regard the fatigue they experienced as being very different to the concept of ‘tiredness’, which is something which everyone experiences on occasions. Furthermore, participants saw sleep as a precious resource, which if disrupted resulted in an exacerbation of their other symptoms.

Participants were already utilising a range of strategies to help them manage their sleep. However, not all of these are necessarily helpful in the long-term, such as daytime naps. These are usually eliminated during a course of CBT-I. Participants also regularly adjusted their sleeping environment and used techniques such as relaxation to help them fall asleep more easily. Poor sleepers tend to make increased efforts to fall asleep, through their thoughts or behaviours, in comparison with good sleepers, who do not seem to need to make such efforts. This may be related to a performance anxiety about sleep, but is likely to fail as the physiological involuntary process of falling asleep cannot be placed under voluntary control (Broomfield and Espie, 2005). Although CBT-I interventions will usually include education about making the bedroom environment comfortable, CBT-I also attempts to reduce ‘sleep effort’.

CBT-I, and the sleep diaries which are used at assessment and during CBT-I therapy, were generally considered acceptable by participants. However, it seems essential that therapists provide a rationale for both the intervention as a whole and individual components which may initially appear counterintuitive to patients. If delivered correctly, however, the educational components of CBT-I will provide a justification for these seemingly paradoxical ideas, such as limiting time in bed and eliminating daytime naps, with the aim of consolidating broken sleep and improving sleep quality. Although a sleep diary is an outcome measure, it was also a useful self-monitoring tool, as it had prepared one patient to make changes to her sleep behaviours.

This study has some limitations. Firstly, patient participants had been living with their diagnosis for an average of 12 years. Newly diagnosed patients may report different experiences. Secondly, the questions within the topic guide are limited. However, the topic guide questions successfully achieved the goal of initiating discussion amongst participants.

This work builds on previous research involving a large number of stakeholders (*n* = 231) and a systematic review of the literature (Hackett, in press; Hackett et al., in press, 2017). Studies by Hackett (in press) and Hackett et al. (in press) both identified sleep disturbances as being problematic for people with PSS. This qualitative piece of work has therefore begun to map the territory of sleep disturbances in PSS and explore potential acceptability and feasibility of future therapy interventions. Future work should build on this by exploring further the specific aspects of sleep disturbances, such as night awakenings, as well as exploring the acceptability of specific modes of intervention delivery such as groups, individual appointments or via a digital interface.

## Conclusion

Sleep may be disrupted in PSS patients, which in turn seems to worsen other symptoms of fatigue and pain. This culminates in a reduced capacity to perform activities during the day. Patients already use strategies to help them manage this symptom, but these may not always be helpful. CBT-I is an intervention which is effective in a range of long-term conditions. This study shows that as long as a rationale is presented, the components of this intervention are likely to be acceptable to PSS patients. However, it should be tailored to the specific requirements of this patient group, whilst recognising the co-morbid symptoms of discomfort and dryness. Occupational therapists should consider using evidence-based CBT-I when addressing disrupted sleep, as the evidence-base for this is greater than for sleep hygiene alone. Successful treatment may have a positive influence on other symptoms and result in improvement in sleep and participation in daytime activities.

## Key findings

- Patients with primary Sjögren’s syndrome experience sleep disruption
- Cognitive behavioural therapy for insomnia is a potentially acceptable therapy
- Improvements in sleep may positively influence other symptoms and improve participation

## What the study has added

This study explored the experience of sleep disturbances in people with primary Sjögren’s syndrome and their partners. Tailored cognitive behavioural therapy for insomnia could improve sleep and daytime participation.
